# Malondialdehyde and anion patterns in exhaled breath condensate among subway workers

**DOI:** 10.1186/s12989-022-00456-z

**Published:** 2022-02-25

**Authors:** Jean-Jacques Sauvain, Maud Hemmendinger, Guillaume Suárez, Camille Creze, Nancy B. Hopf, Valérie Jouannique, Amélie Debatisse, Jacques A. Pralong, Pascal Wild, Irina Guseva Canu

**Affiliations:** 1 Department of Occupational and Environmental Health, Center for Primary Care and Public Health (Unisanté), University Lausanne, Route de la Corniche 2, 1066 Epalinges, Switzerland; 2 Service Santé-Travail, Autonomous Paris Transport Authority (RATP), 88 Boulevard Sébastopol, 75003 Paris, France; 3 Division of Pulmonary Diseases, Geneva University Hospitals and Faculty of Medicine, University of Geneva, Rue Gabrielle Perret-Gentil 4, 1205 Geneva, Switzerland; 4 Division of Research Management, National Research and Safety Institute (INRS), Rue du Morvan, CS 60027, 54519 Vandoeuvre Cedex, France

**Keywords:** Exhaled breath condensate, Underground, Particulate matter, Exposure, Anion, Metabolism

## Abstract

**Background:**

Underground transportation systems can contribute to the daily particulates and metal exposures for both commuter and subway workers. The redox and metabolic changes in workers exposed to such metal-rich particles have yet to be characterized. We hypothesize that the distribution of nitrosative/oxidative stress and related metabolic biomarkers in exhaled breath condensate (EBC) are modified depending on exposures.

**Results:**

Particulate number and size as well as mass concentration and airborne metal content were measured in three groups of nine subway workers (station agents, locomotive operators and security guards). In parallel, pre- and post-shift EBC was collected daily during two consecutive working weeks. In this biological matrix, malondialdehyde, lactate, acetate, propionate, butyrate, formate, pyruvate, the sum of nitrite and nitrate (ΣNO_x_) and the ratio nitrite/nitrate as well as metals and nanoparticle concentrations was determined. Weekly evolution of the log-transformed selected biomarkers as well as their association with exposure variables was investigated using linear mixed effects models with the participant ID as random effect. The professional activity had a strong influence on the pattern of anions and malondialdehyde in EBC. The daily number concentration and the lung deposited surface area of ultrafine particles was consistently and mainly associated with nitrogen oxides variations during the work-shift, with an inhibitory effect on the ΣNO_x_. We observed that the particulate matter (PM) mass was associated with a decreasing level of acetate, lactate and ΣNO_x_ during the work-shift, suggestive of a build-up of these anions during the previous night in response to exposures from the previous day. Lactate was moderately and positively associated with some metals and with the sub-micrometer particle concentration in EBC.

**Conclusions:**

These results are exploratory but suggest that exposure to subway PM could affect concentrations of nitrogen oxides as well as acetate and lactate in EBC of subway workers. The effect is modulated by the particle size and can correspond to the body’s cellular responses under oxidative stress to maintain the redox and/or metabolic homeostasis.

**Supplementary Information:**

The online version contains supplementary material available at 10.1186/s12989-022-00456-z.

## Background

Exposure to ambient particulate matter (PM) is associated with the development and exacerbation of different respiratory and cardiovascular diseases [1, 2]. New evidences suggest that exposure to such pollutants is also able to induce more systemic effects beyond the cardiopulmonary system, e.g. favoring the progress of metabolic diseases like type 2 diabetes or obesity [3]. The full mechanistic understanding of the development for such pathologies is lacking but increasing data suggest that oxidative stress and inflammation are central in explaining these effects. A plausible hypothesis is that the oxidative stress induced by ambient PM exposure modifies the cellular redox homeostasis, resulting in antioxidant depletion, lipid peroxidation and mitochondrial dysfunction [4, 5]. In order to repair and restore the cellular functional processes, a reprogramming of the energy metabolism will occur [6]. Indeed, multiple metabolic pathways have been observed to be modified either in in vitro [7, 8] or in in vivo models [9] after exposure to ambient PM. In these studies, the affected metabolic pathways were related to the metabolism of glucose [10, 11], the tricarboxylic acid cycle as well as glutathione, arginine, proline, nitrogen and lipids metabolism [6]. Due to such multiple and complex inter-relations, a panel of metabolites (and not solely one molecule) should be considered when characterizing and discriminating a pathological state resulting from PM exposure [12].

The pathogenicity from PM exposure has been linked, at least, to their size and chemical composition; particles with smaller aerodynamic diameters are more prone to penetrate deeper into the lung and induce a greater biological activity in sensitive regions such as the alveoli [13]. Ultrafine particles (UFP) (mean aerodynamic diameter < 100 nm) have a high deposition in all regions of the respiratory tract [14]. Beside their chemical variability, related to their sources, ambient PM might also have adsorbing properties for organic compounds or metals, playing the role of a carrier of noxious components to vulnerable regions of the lungs [13]. In order to understand the effect of specific chemicals, it is important to consider model particles enriched with some chemicals. In this frame, underground railways are of special interest as their PM concentration and composition differ strongly from ambient PM [15]. Indeed, PM encountered in subways contain mainly metallic elements, originating from specific processes such as wheel and brake wear or electric arcs. Exposure to PM during subway commuting in different Canadian cities has been reported to contribute to the majority of the personal daily exposure to metals and to 10–20% of the total PM_2.5_ exposure [16]. A similar observation was reported for Milan city, where the contribution of the subway PM on the total mass deposited in the lung was quite important during the winter season [17]. Some of these elements, such as iron, copper, and manganese, might have different oxidation states depending on the biological conditions and be redox-active. Indeed, subway PM possess intrinsic oxidative potential but of variable intensity and mainly associated to copper or nickel but not iron [18, 19]. The possible presence of mixed redox state of metals in these particles, as reported for iron [20] can promote Fenton-like reactions, which induce oxidative damage to biological constituents [21]. Therefore, exposure to subway PM can affect the cell metabolism and particularly, in the mitochondria. Karlsson et al. [22] reported the mitochondria depolarization of human lung epithelial cells A549 to be induced by such PM, suggesting a perturbation in energy storage during the oxidative phosphorylation.

The lung is the main entry portal of PM and by consequence, the place where redox homeostasis perturbations is observed first. It is thus interesting to gain information relative to inflammation or oxidative stress status from this organ. For that purpose, exhaled breath condensate (EBC) is a relevant biological matrix as it is collected non-invasively [23] and allows measuring different biomarkers of exposure and effect [24]. EBC is also proposed as a biological matrix of choice for metabolomics studies [25]. We recently validated a simple method to measure various anions related to metabolism (lactate, acetate, formate, propionate, butyrate, pyruvate) and nitrosative stress (nitrite, nitrate) in EBC [26]. The application of this method to EBC samples collected from workers exposed to soapstone and quartz suggested a modification of the distribution of some of these anions, particularly for quartz exposed workers [26].

In order to evaluate to which extent exposure to subway PM induces metabolic changes in human respiratory system, we made the hypotheses that these particles may have effects on nitrosative/oxidative stress biomarkers (nitrite, nitrate, malondialdehyde) and/or on different metabolism biomarkers (acetate, lactate, formate) measured in the EBC. Our aim was to check these hypotheses based on a longitudinal pilot-study among subway workers followed up during a period of two consecutive weeks, whose exposure was well characterized [27, 28].

## Results

### Anion, MDA, metals and sub-micrometer particle concentrations in EBC

**Fig. 1:**
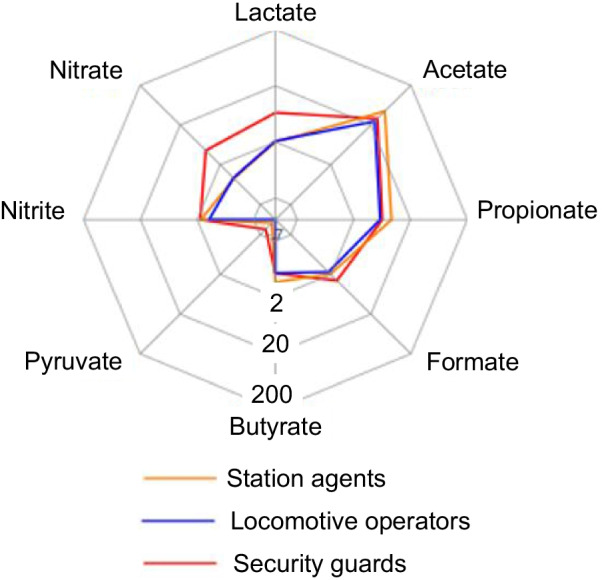
Mean concentrations of the anions [µM] in the EBC of the three professional groups across the day of the week and work-shift

A total of nine workers were enrolled in this pilot study as described in the registered protocol [29]. Three different professional groups were included: station agents, locomotive operators and security guards. Their personal daily exposure to PM_10_, PM_4_, PM_2.5_ and ultrafine fraction as well as their metal content was measured for two consecutive working weeks. In parallel, EBC samples were collected daily pre- and post-shift. Exposure concentrations are described in detail elsewhere [27, 28] and are summarized in Additional file 1: Table S1. Figure 1 (and Additional file 1: Table S2) illustrates the EBC anion concentrations for the three professional groups, averaged across the day of the week and work-shift. The inter-subject variance for the different anions, corresponding to the variance observed during the ten working days, was comprised between 2 and 34% of the total variance. Acetate, propionate and butyrate, corresponding to short chain fatty acids (SCFA), were strongly correlated, with Pearson correlation coefficients comprised between 0.69 and 0.88 (*p*-values < 0.0001 for all correlations), suggesting a similar source (data not shown). The mass proportion of each SCFA anion to their sum was similar for the three professional groups with mean values for acetate/propionate/butyrate of 8/2/0.2 respectively. This distribution is quite different from the typical 3:1:1 ratio observed from microbial fermentation [30]. We noted a moderate correlation between nitrite and formate (Pearson ρ: 0.46, *p* = 0.0001) or nitrite and acetate (Pearson ρ: 0.56, *p* = 0.0001).

MDA could be measured in all professional groups, and most concentrations fell within the concentration range reported in healthy controls, using the same analytical method [31]. Station agents exhibited the highest MDA concentrations (160 ng/L, CI_95%_ = 79–240 µg/L, Additional file 1: Table S2). Iron was a predominant element in the collected underground particles (up to 40.2% for PM_10_, Additional file 1: Table S1), but it could not be consistently quantified in the EBC samples. Only 25% of these samples presented iron levels above the limit of quantification (LOQ) of 1 µg/L and 59% had concentration comprised between LOQ and limit of detection (LOD), set at 0.3 µg/L. The concentration in descending order of the quantified elements in EBC was: Zn > Cu > Ni > Mn. Cu was significantly correlated with Zn and Ni for all professional groups. The averaged concentration of sub-micrometer particles in EBC (using nanoparticle tracking analysis—NTA [26]) was relatively low, comprised between 3.5 and 4.3 × 10^7^ particles/ml, with a mean size distribution between 156 and 174 nm.

### Correlations between the EBC variables

Table 1 presents the Pearson correlation coefficients between the anions and the other variables measured in the 146 EBC samples (MDA, metals and concentration of sub-micrometer particles measured with NTA). Only five variables for anions were considered in this analysis because propionate and butyrate were both strongly correlated with acetate, and pyruvate levels were very low and near the LOD. We observed that lactate was positively and moderately associated with the presence of Cu, Cr, Ni and Zn as well as with the concentrations of sub-micrometer particles in EBC (NTA). Acetate was negatively correlated with most of the variables, but only statistically significant with Cu and Cr. A low but statistically significant positive correlation was also observed between acetate and MDA. Formate was positively and strongly associated with MDA and moderately with Zn. A low but statistically significant positive correlation was observed for the ratio NO_2_^−^/NO_3_^−^ with MDA and negative with Cu and Zn (borderline). No association was observed between MDA and metals in EBC or between MDA and EBC’s sub-micrometer particles (data not shown).

**Table 1:** Correlation* between EBC levels of the different anions with MDA, metals and sub-micrometer particles

	Formate (µmol/L)	Acetate (µmol/L)	Lactate (µmol/L)	ΣNO_x_ (µmol/L)	NO_2_^−^/NO_3_^−^
MDA (ng/L)	**0.53 (0.001)**	**0.29 (0.001)**	0.12 (0.169)	0.06 (0.468)	**0.29 (0.001)**
Cu (µg/L)	0.05 (0.58)	**− 0.21 (0.019)**	**0.47 (0.001)**	0.07 (0.431)	** − 0.23 (0.009)**
Mn (µg/L)	0.11 (0.22)	− 0.19 (0.033)	0.18 (0.048)	0.14 (0.107)	− 0.14 (0.118)
Cr (µg/L)	0.14 (0.134)	**− 0.22 (0.018)**	**0.21 (0.022)**	0.004 (0.970)	− 0.15 (0.100)
Ni (µg/L)	0.03 (0.973)	− 0.07 (0.463)	**0.36 (0.001)**	0.004 (0.968)	− 0.13 (0.150)
Zn (µg/L)	**0.24 (0.008)**	− 0.14 (0.128)	**0.23 (0.010)**	0.18 (0.046)	** − 0.18 (0.044)**
Number concentration (NTA) (#/cm^3^)	− 0.01 (0.945)	− 0.10 (0.241)	**0.32 (0.001)**	− 0.16 (0.083)	0.03 (0.765)
Mean hydrodynamic size (NTA) (nm)	− 0.01 (0.903)	− 0.03 (0.724)	**0.19 (0.038)**	− 0.13 (0.144)	0.10 (0.267)

^*^Pearson correlation coefficients, with *p*-value between bracket. Bold values indicate a statistically significant correlation (*p* < 0.05)

### Dependence of the anion concentration as a function of the professional activity

**Fig. 2:**
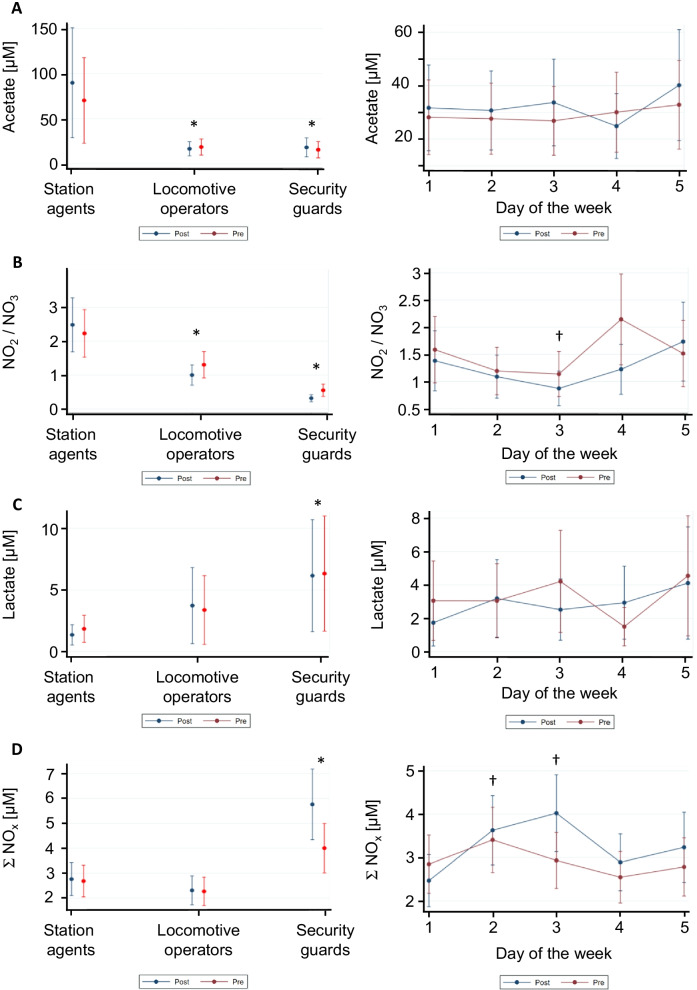
Effect of the professional activity (left panel) and the day of the week (right panel) on the predicted EBC levels of acetate (**A**), for the nitrite/nitrate ratio (**B**), lactate (**C**) and ΣNO_x_ (**D**), either for pre- or post-shift. * denotes a statistically significant difference (*p* < 0.05) with station agents, considered as reference. † denotes a statistically significant difference (*p* < 0.05) with the first day of the week, considered as reference

Results from modelling the anion concentration as a function of work-shift (averaged levels of selected anions for pre- and post-shift are given in Additional file 1: Table S3), day of the week and working group in the mixed model are given in Fig. 2. Compared to the mean concentrations for locomotive operators and security guards, station agents presented a statistically significant higher level of acetate (*p* = 0.001) and NO_2_^−^/NO_3_^−^ ratio (*p* = 0.0001) (Fig. 2A and B). For security guards, lactate (*p* = 0.003) and the sum of nitrite and nitrate (ΣNO_X_, *p* = 0.0001) increased in comparison with station agents and locomotive operators (Fig. 2C and D). In addition, security guards presented the lowest NO_2_^−^/NO_3_^−^ ratio and a higher level of lactate and ΣNO_X_ compared to locomotive operators. We did not observe any clear effect for the day of the week on the anion concentrations, except for the NO_2_^−^/NO_3_^−^ ratio and the ΣNO_X_ (Fig. 2B and D, right panel). For the NO_2_^−^/NO_3_^−^ ratio, a statistically significant decreased ratio was observed on Wednesday (*p* = 0.036) compared to Monday, which was considered as the reference. On the contrary, a statistically significant increase for ΣNO_X_ was determined on Tuesday and Wednesday (*p* = 0.013 and *p* = 0.022 respectively) compared to Monday. MDA level in EBC was the highest for the station agent group (Additional file 1: Table S1) and decreased for locomotive operators and security guards (Additional file 1: Fig. S1), but without reaching a statistical significance (*p* = 0.062).

### Association between exposure and the anion concentrations in EBC

A key question was to know whether the change in concentration of anions during a work-shift (the difference between end of shift and before shift) could be due to the PM exposure, either measured the same day (lag 0), one or two days before (lag 1 and lag 2, respectively). The developed statistical model indicated that the concentration of some anions in EBC quickly changed after exposure (lag 0, Table 2). We observed a positive and significant association (*p* = 0.02) between exposure to PM_4_ and the variation of formate level during the working day. Inhalation of UFP also had a rapid influence on the levels of acetate, ΣNO_x_ and NO_2_^−^/NO_3_^−^. The acetate variation was negatively associated with UFP size (*p* = 0.01) suggesting that the smaller the UFP size, the higher the EBC acetate level at the end of shift. The particle number concentration (PNC) and the lung deposited surface area (LDSA) were also negatively associated with the variation of ΣNO_X_ (*p* = 0.01 and 0.01, respectively). This suggests an inhibitory effect of the PNC and LDSA during the shift on ΣNO_x_. Finally, the variation of the NO_2_^−^/NO_3_^−^ ratio and the PNC were positively associated (*p* = 0.04) for the same day (lag 0). These results indicate that UFP exposure favor the formation of NO_2_^−^ compared to NO_3_^−^.

**Table 2:** Association* between personal exposures and selected anions at lag 0

Personal exposure	MDA_e_/MDA_b_	Formate_e_/Formate_b_	Lactate_e_/Lactate_b_	Acetate_e_/Acetate_b_	ΣNO_x__e_/ΣNO_b_	Nitrite/Nitrate_e_/Nitrite/Nitrate_b_
	Lag 0	Lag 0	Lag 0	Lag 0	Lag 0	Lag 0
Particle number concentration (#/cm^3^)	− 0.06 ± 0.22 (0.77)	0.03 ± 0.08 (0.70)	0.19 ± 0.47 (0.68)	0.14 ± 0.16 (0.37)	− **0.39 ± 0.13 (0.01)**	**0.49 ± 0.24 (0.04)**
UFP size (nm)	− 0.55 ± 0.68 (0.42)	− 0.24 ± 0.25 (0.34)	− 1.32 ± 1.51 (0.38)	− **1.31 ± 0.50 (0.01)**	0.33 ± 0.45 (0.47)	− 0.81 ± 0.79 (0.31)
Lung deposited surface area (µm^2^/cm^3^)	− 0.12 ± 0.25 (0.62)	0.02 ± 0.09 (0.87)	0.17 ± 0.54 (0.75)	0.04 ± 0.19 (0.85)	− **0.48 ± 0.15 (0.01)**	0.54 ± 0.27 (0.06)
PM_2.5_ (µg/m^3^)	− 0.03 ± 0.20 (0.89)	0.05 ± 0.08 (0.54)	− 0.04 ± 0.51 (0.93)	− 0.02 ± 0.18 (0.93)	0.36 ± 0.35 (0.31)	0.40 ± 0.28 (0.16)
PM_4_ (µg/m^3^)	− 0.66 ± 0.48 (0.17)	**0.43 ± 0.18 (0.02)**	− 1.48 ± 1.04 (0.16)	− 0.25 ± 0.37 (0.50)	0.36 ± 0.35 (0.31)	− 0.16 ± 0.59 (0.78)
PM_10_ (µg/m^3^)	− 0.06 ± 0.23 (0.81)	− 0.01 ± 0.09 (0.99)	0.22 ± 0.50 (0.65)	0.14 ± 0.18 (0.44)	0.01 ± 0.17 (0.94)	0.14 ± 0.29 (0.63)

^*^For each pair exposure-anion, the association was evaluated using linear regression with the (log-transformed) daily evolution of anion (ratio post-shift/pre-shift) as dependent variable, the personal exposure as independent predictor variable, and BMI as adjustment variable to control for confounding or effect modification. Bold values correspond to coefficients statistically significantly different from zero. Indice_e_ corresponds to post-shift anions levels whereas indice_b_ corresponds to pre-shift anion levels

Delayed effects (at lag 1, Table 3) resulting from exposure to subway PM on the anions concentrations in EBC were also detected. These effects were mainly associated with the PM mass. A higher EBC lactate concentration was observed at pre-shift compared to post-shift (*p* = 0.01) 24 h after PM_10_ exposure. A similar result was obtained for PM_2.5_ and acetate (*p* = 0.02) and for PM_4_ and ΣNO_x_ (*p* = 0.02). Finally, a statistically significant negative association between the UFP size and the variation of the NO_2_^−^/NO_3_^−^ ratio was observed 24 h after UFP exposure. Concentrations of MDA in EBC was positively associated with exposure to PM_10_ encountered the day before (*p* = 0.05; lag 1, Table 3). At lag 2 (48 h after exposure), no relationship between anion and exposure concentrations was observed.

**Table 3:** Association* between personal exposures and selected anions 24 h after exposure (lag 1)

Personal exposure	MDA_e_/MDA_b_	Formate_e_/Formate_b_	Lactate_e_/Lactate_b_	Acetate_e_/Acetate_b_	ΣNO_xe_/ΣNO_xb_	Nitrit/Nitrat_e_/Nitrit/Nitrat_b_
	Lag 1	Lag 1	Lag 1	Lag 1	Lag 1	Lag 1
Particle number concentration (#/cm^3^)	0.02 ± 0.19 (0.94)	0.03 ± 0.07 (0.60)*	0.06 ± 0.44 (0.89)	0.22 ± 0.18 (0.21)	− 0.04 ± 0.12 (0.76)	0.34 ± 0.27 (0.20)
UFP size (nm)	− 0.97 ± 0.74 (0.19)	− 0.24 ± 0.24 (0.33)	1.93 ± 1.51 (0.20)	− 0.94 ± 0.60 (0.12)	0.68 ± 0.43 (0.13)	**− 2.43 ± 0.88 (0.01)**
Lung deposited surface area (µm^2^/cm^3^)	− 0.08 ± 0.22 (0.74)	0.03 ± 0.08 (0.74)*	0.36 ± 0.52 (0.48)	0.18 ± 0.21 (0.39)	0.02 ± 0.15 (0.88)	0.19 ± 0.32 (0.55)
PM_2.5_ (µg/m^3^)	0.46 ± 0.30 (0.13)	0.01 ± 0.08 (0.86)*	− 0.45 ± 0.45 (0.33)	**− 0.43 ± 0.18 (0.02) †**	− 0.08 ± 0.17 (0.64)	− 0.18 ± 0.31 (0.56)
PM_4_ (µg/m^3^)	− 0.08 ± 0.51 (0.88)	0.17 ± 0.19 (0.37)*	0.29 ± 1.12 (0.79)	− 0.21 ± 0.39 (0.60)	**− 0.88 ± 0.35 (0.02)**	0.94 ± 0.64 (0.15)
PM_10_ (µg/m^3^)	**0.52 ± 0.26 (0.05)**	0.11 ± 0.09 (0.20)*	**− 1.46 ± 0.44 (0.01)**	− 0.24 ± 0.20 (0.24)	− 0.12 ± 0.18 (0.49)	0.39 ± 0.31 (0.21)

^*^For each pair exposure-anion, the association was evaluated using linear regression models with the (log-transformed) daily evolution of anion (ratio post-shift/pre-shift) as dependent variable, the personal exposure as independent predictor variable, and BMI as adjustment variable to control for confounding/effect modification. Bold values correspond to coefficients statistically significantly different from zero. Indice_e_ corresponds to post-shift anions levels whereas indice_b_ corresponds to pre-shift anion levels

^*^BMI statistically significant

^†^Strongly influenced by one extreme point

## Discussion

In this pilot study, we observed that three different professional groups exposed to different subway particulate levels and characteristics presented different patterns of anion in their EBC.

### Effect of exposure on the oxidative stress markers and anion levels in EBC

A detailed description of the workers’ exposures to UFP, PM and metals is given elsewhere [27, 28]. Additional file 1: Table S1 gives an overview of the main exposure levels. Salient results are that station agents had the lowest PM_2.5_ or PM_10_ mass exposure while PNC was the highest among the three groups (20,000 #/cm^3^, CI_95%_ = 16,000–24,000 #/cm^3^). Proportionally, their exposure to fine PM was the highest, with a PM_2.5_/PM_10_ ratio of 0.89. Locomotive operators presented the highest PM_2.5_ and PM_10_ exposures whereas the security guards were the professional group with the largest exposure to the coarse fraction (size range from 2.5 to 10 µm, contributing to about 55% of the PM_10_ mass). Such exposures are similar to the ones measured in different subways in European and Asian metropoles [32]. Exposure differences between the three professional groups were also observed when considering the metal elemental content in the PM. The locomotive operators were exposed to the highest Fe, Zn and Mn concentrations, whereas the security guards had the highest Al level and Cu being as high as for locomotive operators. Nevertheless, when considering metals in EBC, only Cu and Zn could be quantified. The very low Fe concentrations in this matrix suggests a strong sequestration in the lungs for this essential element [27].

In this study, we observed signs of oxidative stress in the worker’s EBC, as measured through MDA quantification, a biomarker of oxidative stress in the airways [33]. The station agents exhibited the highest MDA concentrations, suggesting the presence of oxidative stress in their airways. Interestingly, this group had also the highest PNC exposure. Our finding is in line with in vitro studies showing that transition metals enriched PM, similar to subway PM [15] or residual oil fly ash [34–36], can induce oxidative stress and inflammation. Based on Fig. 2, such a perturbed redox homeostasis could be associated with elevated levels of acetate and a higher ratio NO_2_^−^/NO_3_^−^. On the other side, security guards presented the lowest MDA level in EBC but a statistically significantly higher level of lactate and ΣNO_x_ compared to the two other groups. These different distributions of anions in EBC could be attributed only to their occupational activity. We did not observe a clear effect of the day on the anion concentrations in EBC, indicative of an absence of effect due to a cumulative PM exposure during the week. This suggests a rather constant physiological activity among the different groups or the efficacy of adaptive mechanisms regulating anion homeostasis. As the occupational exposure was different for the three groups, we supposed that the anion variability during a work-shift would be associated with PM exposure, a likely source of these differences in EBC’s metabolites levels. Concentrations of anions in EBC varied depending on the PM fraction and time after exposure. The PNC and LDSA at lag 0 was mainly associated with nitrogen oxides variations during the shift. The negative association between exposure to PNC and the ΣNO_x_ concentrations (Table 2) corresponds to an inhibitory effect of UFP on this variable. This result is coherent with data from Rundell et colleagues [37], who also found a decreased nitrate concentration in the EBC of healthy young subjects exercising during high PNC exposure (> 200,000 #/cm^3^). The observed decrease was attributed either to an inhibition of the nitric oxide synthase (NOS), as observed in rats exposed to diesel particles [38], or to the formation of a peroxynitrite derivative by reaction of NO with superoxide anion. Our additional result indicating a statistically significant association between PNC and the increased proportion of NO_2_^−^ compared to NO_3_^−^ during the work-shift (Table 2) is more in favour of the inhibition of NOS. Indeed, in order to maintain the NO homeostasis, the reductive nitrate-nitrite-NO pathway [39] could be up-regulated, explaining the decreased ΣNO_x_ and the displacement of the ratio NO_2_^−^/NO_3_^−^ in favour of the reduced nitrite form. In coherence with this hypothesis, exposure to UFP has been shown to decrease the levels of nitrate in the blood but not nitrite [40]. Short-term exposure of healthy volunteers to traffic air pollutants has also been shown to induce a transient increase of nitrite levels in EBC [41]. At lag 1, we observed that the PM mass was associated with a decreasing level of acetate, lactate and ΣNO_x_ during the shift. These results suggest a build-up of these anions during the night, in response to the PM exposure of the previous day. Differences in “timing” of action between UFP and micrometer PM has already been reported [42] and attributed to their different fate when deposited in the lungs. In particular, the solubility of metallic particles in the lung lining fluid is different between fine and coarse fractions, with larger particles being less soluble [43]. This could explain the delayed response observed for lactate.

### Relevance of the different anions as biomarkers of effect

The changes in the EBC’s anion concentrations could be the sign of metabolic adaptation in order to face changes in the redox homeostasis resulting from the exposure to metal-rich particles. This additional cellular strategy linking metabolism and antioxidative defense to mitigate ROS and oxidative stress has been recognized only recently for bacteria [44]. We observed that lactate, acetate as well as nitrite and nitrate were the anions mostly affected by exposure to subway PM. The traditional view of lactate as a waste produced during anaerobic conditions has to be reconsidered [45]. Indeed, lactate appears to be a very important metabolite as it links the glycolysis with the aerobic pathways. It is continuously produced during aerobic glycolysis or during stressful conditions and functions as a major energy source for mitochondrial respiration in addition to be an important gluconeogenetic precursor and signaling molecule. Lactate production increases when the biological demand for energy or oxygen exceed their supply [46] and can be considered as a marker of acute lung inflammation [47]. Lactate levels in the EBC of security guards increased compared to locomotive operators and station agents (Fig. 2). In addition, significant correlations were observed between lactate and concentrations of metals or sub-micrometer particles in EBC (Table 1). Taken together, these results are suggestive of an increase in the energy demand to sustain the lung metabolism in security guards. Such energy increase could result from the presence of soluble metals in EBC, which might trigger perturbations in the redox homeostasis. In particular, soluble Cu could be a potent source of H_2_O_2_ or of hydroxyl radical when reducing species like ascorbic acid are present in the milieu [48, 49]. Of note is the higher Cu level measured in EBC of security guards (1.05 µg/L, CI_95%_ = 0.84–1.27 µg/L, Additional file 1: Table S2) compared to the two other professional groups.

Acetate, a short chain fatty acid (SCFA), is a metabolite often detected in EBC due to its high concentration (in the order of tens of micromolar). SCFAs are known to regulate inflammation by acting on leukocytes’ functions and endothelial cells [50]. Our results suggest higher acetate levels for the station agents compared to the two other groups (Fig. 2). This anion is positively correlated with MDA but negatively correlated with Cu and Cr concentration in EBC (Table 1). It is worth mentioning that ROS can contribute to the production of acetate through the oxidative decarboxylation of pyruvate [51]. In that case, pyruvate plays the role of an antioxidant and the resulting acetate metabolite can be used in replacement in the mitochondria, allowing the constant formation of acetyl-CoA, even under hypoxia or other cellular challenges [51]. The positive but rather weak correlation between acetate and MDA (Table 1) could be an argument in favor of this oxidative decarboxylation of pyruvate in presence of ROS. An increase of acetate levels in EBC was reported for different diseases presenting an important inflammatory component like chronic obstructive pulmonary disease [52, 53] or for primary ciliary dyskinesia bronchiectasis [54]. This increase of acetate was attributed to an increase of energy requirement [53, 54]. Nevertheless, metabolomic studies in in vitro assays gave contrasted results. For example, liver [55] or HeLa cells [56] exposed to silica nanoparticles resulted in acetate reduction. Huang et al. [57] also reported a decrease of acetate in A546 cells when exposed to PM_2.5_.

Nitrite (NO_2_^−^) and nitrate (NO_3_^−^) should not be considered as waste products resulting from the oxidation of NO, but rather as a storage pool for NO production, in complement to the NO-synthase (NOS) pathway. Indeed, during hypoxia, acidosis or metabolic stress, when the oxygen-dependent NOS enzyme might be compromised, NO can be produced by reduction of NO_2_^−^ through involvement of haemoglobin, ascorbate or xanthine oxidoreductase among others (nitrate-nitrite-NO reductive pathway, [39]). NO_2_^−^ appears central in controlling vasodilation in hypoxic or in metabolic stress conditions. The increased NO_2_^−^/NO_3_^−^ ratio observed for station agents suggests that for this professional group, the oxidized form (NO_3_^−^) is less favored than NO_2_^−^. In order to explain this observation, we speculate that this increased ratio corresponds to an attempt of the lungs to compensate the locally hypoxic state induced by oxidative stress. Indeed, PM_2.5_ inhalation as well as UFP exposure is associated with a reduced oxygen saturation [58–60] and can induce endothelial dysfunction, particularly by inhibiting the endothelial NO synthase [61]. Such modifications might promote development of pulmonary and systemic inflammation. Magnani et al. [35] showed that mice exposed to metal-coated silica nanoparticles increase their oxygen consumption up to 70%. In that case, vasodilation is necessary to supply a sufficient level of O_2_ for tissue or cell function. In order to maintain the physiologic NO levels needed for regulating the vascular tone, nitrate could be reduced to nitrite, which would be subsequently transformed to NO by NOS-independent pathways. This could explain the observed association between formate and nitrite (Table 1), considering formate as an electron donor for NO_3_^−^ reduction [62]. Finally, the positive correlation between MDA and the ratio NO_2_^−^/NO_3_^−^ (Table 1) suggests that NO_2_^−^ is increased compared to NO_3_^−^ when oxidative stress is present.

ΣNO_x_ appears a promising marker of nitrosative/oxidative stress [63]. ΣNO_x_ for security guards (4.81 µM, CI_95%_ = 3.8–5.8 µM, Additional file 1: Table S2) is consistent with the averaged value of 5.6 ± 5.1 µM, collected from other studies [26]. Compared to these values, station agents and locomotive operators present a lower level of ΣNO_x_ (Additional file 1: Table S2). Saliva contains elevated levels of nitrate, possibly inducing contamination problems during EBC collection. We had to discard all samples from one security guard due to clear salivary contamination resulting in outlier values (NO_3_^−^ levels as high as 218 µM). In this study, security guards were the professional group with the largest exposure to the coarse fraction as mentioned earlier. Such an observation is coherent with results reported by Manney et al. [63], indicating that the coarse fraction of PM measured at central sites of different European cities was the strongest predictor for EBC ΣNO_x_. Knowing that the coarse fraction is often originating from mechanical processes (abrasion, brake and tire wear), such a result points toward the importance of this subway PM fraction in relation to a possible adverse effect on the respiratory system.

Exposure to micro or nano-sized ZnO particles [64, 65] or welding fumes containing zinc and/or copper [66] has been shown to induce an acute phase response through the increase of serum amyloid A and C-reactive proteins in blood of healthy exposed volunteers. Elevation of these two proteins in blood is associated with an increased risk of cardiovascular diseases. By analogy to these reports, and considering the presence of Cu and Zn in EBC as well as the modified pattern of anions (mainly ΣNO_x_) in our subway workers, adverse cardiovascular effects might be expected in this population. Whereas we did not sample blood in our study due to its invasive nature, blood should be considered in further research regarding possible effects of subway PM exposure on cardiovascular system.

### Strengths and limitations

One of the main strengths of this study is that the individual exposure to different PM fractions has been thoroughly characterised with a simultaneous collection of EBC over the two-week period. This allowed us to look precisely to the relationships between exposure and metabolite levels. In addition, we used validated methods for the analysis of these different metabolites [69, 70]. This is a prerequisite for proposing EBC as a relevant matrix for future clinical studies [67]. Finally, an approach combining multiple biomarkers in EBC is mandatory, as multiple and intricated metabolic pathways are often influenced by exposure to different environmental stressors [6]. For example, the metabolomics approach usually outperforms the discrimination between asthmatic and non-asthmatics compared to conventional clinical tests using either exhaled NO or standard spirometry [68]. The additional interest to focus on a pattern of different metabolites in EBC relates to the fact that it gives insight into the actual functional status of the lungs and comparison with basal levels could indicate atypical physiological status [6].

Nevertheless, the selected anions in this study were related only to nitrosative stress and glycolysis, narrowing the number of considered pathways when compared to more sophisticated metabolomics techniques. It is the reason why such comprehensive techniques should be involved in complement to the more specific and focussed approach we used in this pilot study. The issue of a low number of participants included was partially resolved by the longitudinal design of the study with a continuous personal exposure measure over the work-shift and twice a day measures of the outcomes for each participant. Indeed, we expected a decreased inter-individual variability, improving by this way the possibility to observe modifications of the metabolites levels in EBC. However, we acknowledge that all comparisons of job groups, even when statistically significant, lack robustness. Moreover, a gender effect might be present as the station agents group consisted only of women, in contrary to both other groups. That is why the results pertaining to the comparison of job groups have to be considered with caution and will need further confirmation. For that purpose, an epidemiological study including about 300 volunteers belonging to these three professional groups is in progress in the same company, with the intended aim to demonstrate the usefulness of the different selected anions from this pilot study [29]. A final issue is the multiplicity of comparisons. We did not provide Bonferroni-type adjusted p-values given that such adjustments rely on the number of tests made which can be easily manipulated. We prefer to consider our results as exploratory and a statistical significant result as a flag for the reader of a possible real effect.

## Conclusion

In this pilot study, we found that three different professional groups exposed to subway particulate levels and occupational conditions presented specific patterns of anions in their EBC. Nitrogen oxides (nitrite/nitrate ratio and ΣNO_x_) as well as acetate and lactate appeared the most modified metabolites after exposure to subway PM. We also observed that these changes are modulated by the PM size, with UFP and coarse particles inducing different responses. Such changes are suggestive of cellular strategies to maintain the redox and metabolic balance when under oxidative stress.

The combined measurement of these anions in EBC could be helpful to investigate the cardiopulmonary effects resulting from exposure to subway PM on workers and the general population.

## Material and methods

### Study design and participants

This longitudinal pilot-study belongs to the broader Respiratory disease Occupational Biomonitoring Collaborative Project (ROBoCoP project), which research protocol has been described elsewhere [29]. The aims of this pilot study were to: 1. understand the suitability of different candidate biomarkers for biomonitoring of workers exposed to metallic PM; 2. to select the most relevant biomarkers for COPD or asthma detection in a larger epidemiological study of subway workers. Volunteers were recruited based on their workplace attribution to the subway line 7. This line is completely underground with PM concentrations corresponding to the worst case scenario. Three different professional groups were recruited, each composed of three healthy nonsmokers: station agents (women only); locomotive operators and security guards (both composed of men only). A detailed description of their activities is available in [27]. We measured exposure parameters and collected biological matrices by job type during two consecutive weeks for each group.

### Exposure to particulate matter and analysis

Worker’s personal exposure to different PM fractions (UFP, PM_2.5_, PM_4_, PM_10_) and total metal concentration was determined for all nine volunteers during two consecutive weeks (excluding the weekend) [27]. Briefly, active personal sampling for PM was achieved using Teflon filters connected to a pump with flow set at 4 or 10 L/min, depending on the collected fraction. After standard gravimetric analysis of the filters, they were digested in acids and the resulting solution analyzed by inductive coupled plasma mass spectrometry (ICP-MS; ICap TQ, Thermo Scientific, Switzerland) for 11 elements (Al, As, Ba, Cd, Cr, Cu, Fe, Mn, Ni, Pb and Zn). A direct-reading particle counter instrument (DiscMini, Testo, Switzerland) was used for personal UFP measurement.

### EBC collection and analysis

Pre- and post-shift EBC samples were collected on a daily basis using a portable collection device (Turbo-DECCS, Medivac, Parma, Italy) set at – 10 °C. Sampling took place in a clean room, located either at the subway station Porte de la Villette or at Gare de Lyon and following the recommendations of the American Thoracic Society and the European Respiratory Society Task Force [23]. None of participants declared drinking tea or coffee an hour before EBC collection. A total volume of 2–3 mL of EBC per participant was collected during the 20 min sampling. Immediately after collection, EBC sample was aliquoted in the same room. For anion analysis, 100 µl of the collected EBC was transferred into conical plastic vials (300 µl, Macherey Nagel, Düren, Germany) whereas 250 µl of EBC was aliquoted in vials (1.5 ml, Macherey Nagel, Düren, Germany) for MDA analysis. Aliquots were frozen at − 80 °C until analysis.

Anions associated to nitrosative stress (NO_2_^−^ and NO_3_^−^ as stable end-product of NO oxidation) or to metabolism (lactate, acetate, propionate, butyrate, formate and pyruvate) were analyzed in EBC following a validated method [26]. Briefly, 10 µl of the EBC sample was injected without any treatment into a Dionex ICS 5000 + ion chromatograph, equipped with an analytical column IonPac AS11-HC250 mm, 4 µm (ThermoFisher Scientific, Ecublens, Switzerland) and a conductivity detector. The low LOD, comprised between 0.07 and 0.58 µM (depending on the analyte) allowed the quantification of all these anions in all samples.

MDA is a stable end-product of lipid peroxidation and is considered as a marker of oxidative stress [33]. This molecule was measured in the EBC sample using dinitrophenyl hydrazine (DNPH) as derivatisation agent. Briefly, the validated method [69] consisted in adding 50 µl of a 2 mM DNPH solution to 125 µl of EBC sample, containing MDA-d_2_ as internal standard. The reaction mixture was heated to 50 °C during 2 h, cooled to ambient temperature than directly injected into a LC–MS/MS instrument. About 19% of the samples had MDA concentrations below the LOD of 70 pg/ml.

Copper (Cu), manganese (Mn), nickel (Ni), chrome (Cr) and zinc (Zn) as well as the number concentration of sub-micrometer particles in the EBC samples were determined by ICP-MS technique (metals) and nanoparticle tracking analysis (NTA) respectively. Details for theses analytical methods can be found in [27] and [70], respectively. The observed LOD was 0.003 µg/L for Cr, Ni and Mn, 0.07 µg/L for Cu and 0.3 µg/L for Zn and Fe. The LOD for the NTA was 2.7 × 10^7^ particles/ml.

### Data management and statistical analysis

One of the security guards had a high salivary production during the EBC collection, which contaminated some of his samples. This resulted in a very high NO_3_^−^ concentration in these EBC samples. We considered this worker as an outlier and therefore these samples were excluded from the statistical analyses reported in this paper.

Individuals’ anion concentrations, nitrite/nitrate ratio as well as the sum of nitrite and nitrate (ΣNO_x_) were first log-transformed. In a second step, these variables were analyzed using linear mixed effects models with the subject ID as a random effect. For each of these outcomes, we fitted a model including the job group, the day of the week (in order to detect a possible cumulative effect over the week) and the shift (pre- vs. post-shift). All pairwise interactions were tested between these three factors. We further adjusted on the body mass index (BMI) in order to account for a possible effect of this factor on the metabolism. In a third step, pairwise Pearson correlation coefficients were computed for the variable measured in EBC samples (anions, MDA, metals, sub-micrometer particles). Given that these substances were all measured within the same EBC samples, these correlations coefficients did not take into account any other variable. This enabled, on the one hand, to assess the presumed associations related to nitrosative/oxidative stress or metabolism, and, on the other hand, to highlight potential associations between exposure biomarkers (metals, sub-micrometer particles) and effect biomarkers (MDA, anions).

Lastly, the within-day evolution of EBC anion concentrations was characterized for each anion as the ratio of the post-shift and the pre-shift concentrations. These (log-transformed) ratios were then analyzed using a linear mixed effect model with the participant ID as a random effect variable. Independent variables were the external exposure measurements conducted in the workplace (PM_10_, PM_2.5_, airborne metal concentration and PNC) obtained during either the same (lag 0) or previous (lag 1) EBC collection day. For lag 1, the first day of the working week was excluded. Standard checks for heteroskedasticity and outliers were performed. These led to the exclusion of the subject mentioned at the beginning of the paragraph.

## Supplementary Information

**Additional file 1: Supplemental Table S1**. Average exposure level of the three different professional groups.** Supplemental Table S2**. Geomtric concentration of the selected variables in EBC samples collected during the two working weeks for the three professional categories.** Supplemental Figure S1**. Effect of the professional activity on the predicted EBC levels of MDA.** Supplemental Table S3**. Averaged EBC concentration of selected anions in pre- and post-shift for all volunteers.

## Data Availability

The datasets used and/or analyzed for the current study are available from the corresponding author on reasonable request.

## Abbreviations

CI_95%_: 95% Confidence interval
Cr: Chromium
Cu: Copper
EBC: Exhaled breath condensate
Fe: Iron
H_2_O_2_: Hydrogen peroxide
ICP-MS: Inductively coupled plasma mass spectrometry
LDSA: Lung deposited surface area
LOD: Limit of detection
LOQ: Limit of quantification
MDA: Malondialdehyde
Mn: Manganese
Ni: Nickel
NO: Nitric oxide
NO_2_^−^/NO_3_^−^: Ratio of nitrite to nitrate
NOS: Nitric oxide synthase
ΣNO_X_: Sum of nitrite and nitrate
NTA: Nanoparticle tracking analysis
PM: Particulate matter
PNC: Particle number concentration
RoBoCoP: Respiratory disease occupational biomonitoring collaborative project
ROS: Reactive oxygen species
SCFA: Short chain fatty acids
UFP: Ultrafine particles
Zn: Zinc
