# Supplementary material for: Malondialdehyde and anion patterns in exhaled breath condensate among subway workers
Source: Part Fibre Toxicol. 2022 Feb 25;19:16. doi: 10.1186/s12989-022-00456-z (PMC8876786; doi:10.1186/s12989-022-00456-z)
Supplement: Supplementary file 1 — Additional file 1: Supplemental Table S1. Average exposure level of the three different professional groups. Supplemental Table S2. Geomtric concentration of the selected variables in EBC samples collected during the two working weeks for the three professional categories. Supplemental Figure S1. Effect of the professional activity on the predicted EBC levels of MDA. Supplemental Table S3. Averaged EBC concentration of selected anions in pre- and post-shift for all volunteers. [file 12989_2022_456_MOESM1_ESM.docx]

**Supplementary material**

**Malondialdehyde and anion patterns in exhaled breath condensate among subway workers.**

Jean-Jacques Sauvain^1^*, Maud Hemmendinger^1^, Guillaume Suárez^1^, Camille Creze^1^, Nancy B. Hopf^1^, Valérie Jouannique^2^, Amélie Debatisse^2^, Jacques-André Pralong^3^, Pascal Wild^4^, Irina Guseva Canu^1^

**Exposure levels of the three professional categories.**

Table S1: Average particulate matter concentration (with 95% confidence interval values into bracket) of the different fractions (ultrafine, PM_2.5_, PM_4_ and PM_10_) measured for the three different professional groups, as well as the proportion of different elements (iron, copper, manganese and zinc) in the PM_10_ and PM_2.5_ fraction. UFP: Ultrafine particle; LDSA: Lung deposited surface area. Bold variables indicate a statistically significant difference between the different groups (p<0.05).

|  | Station agents  (n=30) | Locomotive operators (n=9) | Security guards  (n=18) |
| --- | --- | --- | --- |
| **PM_10_ [µg/m^3^]** | 49 [35-63] | 174 [92-257] | 81[54-109] |
| **PM_4_ [µg/m^3^]** | 69 [59-80] | 100 [75-124] | 88 [73-102] |
| **PM_2.5_ [µg/m3]** | 45 [31-60] | 140 [77-203] | 46 [30-63] |
| PM_2.5_/PM_10_ | 0.89 | 0.82 | 0.45 |
| **UFP Number [#/cm^3^]** | 20’000 [16’000-24’000] | 15’000 [12’000-17’000] | 8’900 [7’000-11’000] |
| **UFP Size [nm]** | 37 [35–39] | 50 [47–52] | 49 [46–52] |
| **UFP LDSA [µm^2^/cm^3^]** | 38 [32–45] | 40 [34–46] | 24 [19–29] |
| **Ratio Fe/PM_10_ [%]** | 3.1 [2.2-4.0] | 25.7 [11.3-40.2] | 13.2 [8.0-18.5] |
| **Ratio Fe/PM_2.5_ [%]** | 2.1 [1.6-2.7] | 11.1 [6.3-15.8] | 8.4 [5.8-10.9] |
| Ratio Cu/PM_10_ [%] | 0.17 [0.09-0.24] | 0.12 [0.06-0.18] | 0.21 [0.14-0.29] |
| Ratio Cu/PM_2.5_ [%] | 0.28 [0.17-0.38] | 0.17 [0.01-0.32] | 0.13 [0.04-0.21] |
| Ratio Mn/PM_10_ [%] | 0.13 [0.06-0.20] | 0.24 [0.12-0.37] | 0.16 [0.09-0.22] |
| **Ratio Mn/PM_2.5_ [%]** | 0.14 [0.05-0.22] | 0.12 [0.07-0.17] | 0.24 [0.15-0.33] |
| **Ratio Zn/PM_10_ [%]** | 0.95 [0.77-1.13] | 0.59 [0.38-0.81] | 0.44 [0.33-0.56] |
| Ratio Zn/PM_2.5_ [%] | 0.59 [0.45-0.73] | 0.45 [0.28-0.63] | 0.67 [0.48-0.85] |

**Averaged EBC level of the considered variables**

Table S2: Estimated geometric concentration ± 95% confidence interval into bracket of the selected variables in EBC samples collected during the two working weeks for the three professional categories. The mixed-model used for this estimation considered only the profession as explicative variable. Bold variables indicate statistically differences between the three professional groups (p<0.05).

|  | Station agents (n=57) | Locomotive operators (n=52) | Security guards (n=36)^a^ |  |
| --- | --- | --- | --- | --- |
| MDA [pg/ml] | 160 [79-240] | 100 [50-160] | 61 [23-99] |  |
| **Acetate [µM]** | 44.6 [20.1-69.1] | 25.2 [11.3-39.1] | 14.9 [4.8-24.9] |  |
| **Propionate [µM]** | 9.28 [5.65-12.92] | 5.83 [3.530-8.13] | 3.81 [1.97-5.64] |  |
| **Butyrate [µM]** | 1.12 [0.84-1.41] | 0.78 [0.58-0.98] | 0.46 [0.31-0.61] |  |
| Formate [µM] | 1.95 [1.61-2.29] | 1.73 [1.43-2.04] | 2.00 [1.57-2.43] |  |
| **Lactate [µM]** | 2.04 [0.92-3.16] | 2.10 [0.93-3.28] | 5.74 [1.84-9.64] |  |
| Pyruvate [µM] | 0.005 [-0.01-0.02] | 0.001 [-0.02-0.003] | 0.002 [-0.005-0.009] |  |
| Nitrite [µM] | 1.71 [1.35-2.06] | 1.23 [0.97-1.49] | 1.25 [0.93-1.57] |  |
| **Nitrate [µM]** | 0.90 [0.72-1.08] | 0.93 [0.74-1.12] | 3.09 [2.31-3.87] |  |
| **ΣNOx [µM]** | 2.71 [2.25-3.16] | 2.28 [1.88-2.67] | 4.81 [3.80-5.82] |  |
| **NO_2_^-^/NO_3_^-^** | 1.90 [1.46-2.34] | 1.32 [1.00-1.64] | 0.40 [0.29-0.52] |  |
| Cu [µg/L] | 0.88 [0.72-1.03] | 0.84 [0.71-0.98] | 1.05 [0.84-1.27] | |
| Mn [µg/L] | 0.13 [0.11-0.15] | 0.13 [0.11-0.15] | 0.15 [0.12-0.18] | |
| Ni [µg/L] | 0.30 [0.23-0.37] | 0.23 [0.18-0.28] | 0.23 [0.16-0.29] | |
| **Cr [µg/L]** | 0.022 [0.017-0.028] | 0.038 [0.030-0.046] | 0.036 [0.026-0.045] | |
| Zn [µg/L] | 9.6 [7.7-11.4] | 7.1 [5.8-8.4] | 9.0 [7.0-11.0] | |
| NTA number [#/cm^3^]*10^7^ | 2.8 [2.1-3.4] | 3.8 [3.0-4.6] | 2.6 [1.9-3.4] |  |
| NTA mean size [nm] | 135 [128-143] | 144 [136-152] | 132 [123-141] |  |

^a^: one outlier removed.

Figure S1: Effect of the professional activity on the predicted EBC levels of MDA.

**Averaged EBC level of the selected anions pre- and post-shift**

Table S3: Averaged EBC concentration ± standard deviation of selected anions in pre- and post-shift for all the volunteer. The number of measurement is given in bracket.

|  | Pre-shift | Post-shift |
| --- | --- | --- |
| Acetate [µM] | 41.3 ± 28.4 (71) | 33.4 ± 20.0 (73) |
| Lactate [µM] | 6.8 ± 8.9 (66) | 5.7 ± 5.4 (68) |
| ΣNOx [µM] | 4.3 ± 7.2 (72) | 3.1 ± 1.7 (73) |
| NO_2_^-^/NO_3_^-^ | 1.54 ± 1.25 (72) | 1.73 ± 1.40 (73) |
